# The Prothrombotic Phenotype of Thirdhand Electronic Cigarette Exposure is Sex Independent and Involves Systemic Mediated Effects on Platelet Function: Evidence from a Mouse Model

**DOI:** 10.1007/s12012-026-10093-z

**Published:** 2026-02-17

**Authors:** Shelby S. Umphres, Ahmed B. Alarabi, Hamdy E. A. Ali, Shahnaz Qadri, Fadi T. Khasawneh, Fatima Z. Alshbool

**Affiliations:** 1 Department of Pharmacy Practice, Irma Lerma Rangel College of Pharmacy, Texas A&M University, 1010 W Avenue B, Kingsville, TX 78363 USA; 2 Department of Pharmaceutical Sciences, Irma Lerma Rangel College of Pharmacy, Texas A&M University, 1010 W Avenue B, Kingsville TX 78363, USA

**Keywords:** Platelets, Cytokines, Thirdhand exposure, E-cigarettes, Thrombosis

## Abstract

Electronic cigarettes (e-cigs) are major contributors to inflammatory-mediated responses, which are implicated in a vast array of pathophysiological conditions, including cardiovascular disease (CVD). More recently e-cigs have been recognized as a source of thirdhand exposure (THEC); the process by which expelled toxins settle on materials (i.e., carpets, curtains, clothes etc.) and undergo chemical reactions, rendering them more harmful overtime. Herein, mice were exposed to THEC for four months, and platelet reactivity, systemic mediated effects on platelet function, and cytokine expression profiles were analyzed in both sexes. Our data revealed a hyperactive platelet phenotype as determined by shortened bleeding and occlusion times, enhanced platelet aggregation, and dense granule secretion with no significant difference between males and females. Cytokines, amongst other inflammatory molecules, are well documented mediators by which platelet function is modulated and they also enhance susceptibility to CVD. To this end, and to elucidate the mechanism by which platelet reactivity was augmented, washed platelets that were exposed only to clean air (CA) and resuspended in THEC exposed plasma, displayed significantly increased platelet aggregation, dense granule secretion, and p-selectin expression. Indeed, this data suggests that THEC exposure elicits a systemic effect, enhancing platelet response, and was further validated by a dysregulated cytokine profile using plasma, free of platelets, in a sex-dependent manner. Collectively and for the first time, we highlight that both males and females are at similar risk of THEC-mediated prothrombotic phenotype, which is underlined-at least in part- by an indirect systemic effect of exposure on platelet reactivity that involves changes in the cytokine profile. These findings underscore this form of exposure as a threat to cardiovascular health.

## Introduction

There is well documented evidence highlighting the health risks that are associated with tobacco use, specifically in the context of cardiovascular disease (CVD), the leading cause of death worldwide [1]. In fact, most CVD related deaths are due to thrombotic events such as stroke and heart attack. Notably, platelets play a major role in the hemostatic process but can also greatly contribute to an increased risk for CVD, when activated under pathophysiological conditions [2]. The mechanisms by which platelet activation occurs include several complex pathways which are further enhanced, particularly in the event of injury, illness, and environmental insults [3]. In light of the fact that the use of electronic cigarettes (e-cigs) is on the rise, ongoing studies have been providing greater insight into their negative health impact. These studies have highlighted pathophysiological outcomes on CV health, in the context of direct exposure, which is the delivery of aerosolized vapor directly to the lungs of the user [4, 5]. To this end, data from our laboratory has shown that direct e-cig exposure can significantly heighten the risk of thrombogenesis-based disease states and promote platelet hyperactivity [6, 7]. The popularity of e-cigs necessitates that the characterization of their negative effects is expanded beyond the direct exposure, which involves indirect exposure to vape expelled toxins onto household materials (i.e., carpet, curtains, couches, etc.); also known as thirdhand e-cig (THEC) exposure [8]. These toxins undergo chemical reactions/oxidation over time and form strong radicals that have the capacity to initiate damage to proteins, lipids and DNA [9, 10]. A recent report from our laboratory- utilizing a mixed gender mouse model- provided the first evidence that THEC exposure is indeed a new health threat via promoting a prothrombotic phenotype [11]. Although some progress has been made towards highlighting the CV related risk of THEC exposure, many questions remain unanswered, some of which require more comprehensive studies that include both sexes.

It is noteworthy that studies examining the sex-dependent CV effects of cigarettes and e-cigs continue to evolve and suggest that sex differences do exist [12, 13]. To this end, smoking increases the risk of CVD more in females compared to males [12]. It was also found that women are more susceptible to platelet damage caused by smoking [14]. However, no differences in platelet reactivity were observed between males and females with the use of heat-not-burn cigarettes [15]. Though it is not concretely established, it has been shown to some extent that females are more susceptible to impaired CV functions due to e-cig usage in comparison to males [16]. In terms of thirdhand exposure, we have previously reported that thirdhand smoke (THS) exerts sex-dependent effects on occlusive CVD [17], including under in utero/maternal settings [18]. However, the role of sex in THEC exposure mediated negative effects remains ill-defined and has yet to be investigated.

Systemic/in-direct effects on platelet function in relation to THEC exposure cannot be excluded/should also be considered. In this connection, platelets can be activated by molecules produced or released (e.g., cytokines & chemokines) from other cell types, which are considered a source of inflammation and hemostatic imbalance [19]. Indeed, there is an abundance of evidence-based data of inflammatory responses caused by smoking [20–22]. Importantly, it has been shown that direct e-cig exposure produces pro-inflammatory molecules, such as IL-6, CXCL8, ROS, and TNFα, which could be a result of heating the e-liquid, thereby potentiating a prothrombotic state through endothelial dysfunction, oxidative stress, as well as effects on platelet activation and coagulation [5]. To this end, we aim to explore the sex-specific effects of the THEC mediated prothrombotic phenotype and modulation of platelet function. Moreover, we will characterize the mechanism underlying THEC effects by exploring the systematic effects on platelets and seeking to identify the impact on cytokines, in a sex-dependent manner.

## Materials and Methods

### Materials and Reagents

Exposure materials including muslin natural fabric (cotton) were purchased from Amazon, the K9975 dresden upholstery (polyester, cotton, and olefin) from Kovi Fabrics, the Nourison spectrum carpet (rayon, wool, and nylon) from Bed Bath and Beyond, and the e-liquid and tanks were from Vapor Chef (Bristol, PA, USA). The Mouse/Rat Cotinine ELISA kit was purchased from Calbiotech (El Cajon, CA, USA). Agonists and the aggregation supplies such as collagen, luciferase, stir bars, and cuvettes were all purchased from Chrono-Log Corporation (Havertown, PA, USA). Coverslips were from VWR (USA), and cell mounting media with TRITC fluorophore conjugated phalloidin (catalog # H1200) was from Vector laboratories, Inc. (Newark, CA, USA); which were used for staining actin in the platelet spreading assay. Flow cytometry materials such as APC anti-mouse CD41 (catalog # 133914) was purchased from BioLegend® (San Diego, CA, USA), and FITC conjugated P-selectin (catalog # 553744) was purchased from BD Biosciences (San Diego, CA, USA). The Proteome Profiler Mouse XL Cytokine Array was purchased from R&D Systems (Minneapolis, MN, USA). Other materials including ferric chloride (FeCl_3_), apyrase, and fibrinogen were from Sigma-Aldrich (St. Louis, MO, USA), whereas PGI_2_ was purchased from Caymen Chemicals (Ann Arbor, MI, USA). All other reagents used were of analytical grade.

### Exposure Protocol

Simulating real-world exposure conditions, we employed our established thirdhand exposure protocol, as previously described [11]. Briefly, a custom e-vapeTM vapor inhalation system from La Jolla Alcohol Research, Inc. was used to expose two sets of materials on an alternating weekly basis; with each set consisting of 3 pieces of fabric (7.5 × 5.5 in), 2 pieces of carpet (2 × 2 in), and 1 piece of upholstery (12 × 3.5 in). Materials were hung on racks inside the inhalation chamber (volume is 3,081 in^3^) to ensure an even distribution of e-cig vape. Menthol flavored Absolute 0 e-liquid (Vapor Chef) containing 18 mg of nicotine and 30/70 propylene glycol/vegetable glycerin (PG/VG) was placed inside a TFV8 Big Baby tank, which was used to generate the vape, with voltage (V) set at 5 V and resistance set at 0.4 ohms. Each day, the materials were exposed to one round of 400 puffs with a 3 s puff duration, 30 s between each puff, and a 50 mL puff volume. The system airflow was maintained at 1 L/minute. This was repeated for a total of 5 days with each set of materials exposed on consecutive weeks. Male and female mice were housed as described in the animal’s section and lived with either clean air (CA) or thirdhand e-cigarette (THEC) exposed materials for a duration of 4 months, with full access to the materials. By employing two sets of materials, this approach ensured that a fresh set of fully exposed materials were added to the cages each week, as the other set (old materials) are/were re-exposed. After four rounds of exposure to the same set, a new set of materials were utilized to ensure there is exposure to both fresh and aged THEC as in real-life settings.

### Animals

Male and female C57BL/6 J mice were purchased from Jackson Laboratories (Bar Harbor, ME, USA) and same sex mice were housed 5 mice/cage on flow racks that were maintained at 10–15 air changes per hour. Each cage had a volume of 458.6 in^3^. Standard care and conditions were provided to the mice, including 12/12 light/dark cycle, at 24 °C with food and water access ad libitum. In addition to these conditions, mice were housed with either the CA or THEC exposed sets of materials beginning at 6 weeks of age. Experiments were conducted following the 4-month exposure period and all experimental protocols were approved by the Texas A&M University Institutional Animal Care and Use Committee.

### Blood and Platelet Rich Plasma (PRP) Collection

Blood was collected via cardiopuncture after mice were anesthetized with 2–5% isoflurane (SomnoFlo System). Samples were combined with 50 µL of sodium citrate (3.8%) to prevent coagulation. Citrated blood was centrifuged at 180 g for 11 min at 25 °C using a Thermo Scientific Sorvall Legend XTR system, and the PRP was collected and used for further experimentation.

### Blood Cell Counts

Whole blood from individual mice was collected and platelets, and all other blood cell counts were conducted using a HEMAVET® 950FS Multi-species Hematology System.

### Cotinine ELISA

Blood was collected from individual mice via cardio puncture, without sodium citrate, and left to clot. Blood was spun at 3,000 g for 15 min at 25 °C and the serum was collected. Individual serum samples were run using a Mouse/Rat Cotinine ELISA kit per the manufacturer’s protocol.

### Tail Bleeding Time Assay

Mice were anesthetized and placed on a homeothermic blanket maintained at 37 °C. A 5 mm segment of the tail was transected and immediately placed in a 0.9% sodium chloride solution, and the time to bleeding cessation was measured. To prevent excess bleeding and for the purpose of statistical analysis, the bleeding cut off time was considered to be 10 min. This method was performed as previously described [24, 25].

### In vivo* FeCl*_***3***_*-Induced Thrombosis Model*

Mice were anesthetized with 2.5% avertin (IP injection) and placed on a 37 °C homeothermic blanket. The left carotid artery was isolated and cleaned and the blood flow baseline was measured for 1 min using a 0.5-mm micro-flow probe (Transonic Systems Inc.). The artery was then dried to prevent any dilution, and a 1 mm filter paper soaked with 7.5% FeCl_3_ was placed directly on the artery for 3 min. After the injury was induced by FeCl_3_, the filter paper was removed, and the time to occlusion was measured using the flow probe. A 15-min occlusion time was considered the cut off time for the purpose of statistical analysis.

### In Vitro Platelet Aggregation and Dense Granule Secretion

Blood was pooled from 5–8 mice in the CA exposed group or 5–8 mice in the THEC exposed group, and this was done for each sex (males and females) separately. PRP was then isolated and stimulated with 1.25 µg/mL of the agonist collagen. Dense granule secretion was also determined using 12.5 µL of luciferase in each of the samples. Both platelet aggregation and dense granule secretion were measured using a model 700 Chrono-Log aggregometer, and each experiment was conducted at least 3 times. Platelet concentrations were fixed to 175,000/µL and PRP volume was adjusted accordingly in a total of 250 µL in each cuvette for each sample. The remaining volume was made up using Tyrode’s buffer (pH 7.4). The % maximal aggregation values, which represent the highest level of aggregation achieved after an agonist is added, were extracted from Aggolink8 software and averaged.

### Washed Platelet Preparation

PRP was collected as previously described [25] from both CA exposed and THEC exposed groups. Samples were incubated with 10 ng/mL of PGI_2_ and 0.37 U/mL of apyrase and spun at 400 g for 10 min at 25 °C. The supernatant was discarded, and the pellet was resuspended in Tyrode’s buffer (pH 6.5), containing 10 ng/mL of PGI_2_, 0.37 U/mL of apyrase, and 0.5 M EGTA. Samples were spun again at 400 g for 10 min at 25 °C and the supernatant was removed. The pellet was resuspended in Tyrode’s buffer (pH 7.4) and washed platelets were counted as previously mentioned. All platelet concentrations were accordingly adjusted for both CA and THEC prior to experimentation, to ensure equal number of platelets are used.

### Platelet Spreading

Platelet spreading was done as previously described [26]. Blood was pooled from 5–8 mice in the CA exposed group or 5–8 mice in the THEC exposed group from each sex separately, and washed platelets were prepared and counted as described above. The platelet counts were adjusted, and 100,000 k/µL of platelets were added to fibrinogen-coated coverslips in triplicates for each of the groups per the corresponding sex, and incubated at room temperature for 10 min. After the incubation period, unbound platelets were removed using Tyrode’s buffer (pH 7.4). The bound platelets were then activated with 0.005 U/mL of thrombin and incubated for an additional 10 min before being fixed with 4% paraformaldehyde. Following fixation, the platelets were washed with PBS, permeabilized with 0.5% saponin, and incubated for 15 min. Cell mounting media containing phalloidin conjugated-TRITC fluorophore was added to the fixed platelets, which were mounted on a microscopy slide using transparent microscopy-grade nail polish. Confocal microscope (Nikon Eclipse i2) 60X Apo 1.4 objective lens was used for imaging, and field of view (FOV) was 2048*2048 pixels. LASER 540 nm was used to excite the TRITC fluorophore to observe actin in platelets. The ImageJ software was utilized to process images and quantify the number of activated platelets. The platelets with polymerized actin were considered activated. Actin polymerization in platelets was confirmed by forming skeletal structures, which were further confirmed by using images generated from the Shape Index Map tool. For analysis, the number of activated platelets was quantified from 10 images taken at different fields in each slide, and 3 slides were prepared per group. A total of 30 images for each group were used in statistical analysis.

### Aggregation and Dense Granule Secretion in Resuspended Platelets

Blood was pooled from 5–8 mice in the CA exposed group or 5–8 mice in the THEC exposed group, from each sex separately. Each pooled group was further divided into two groups and this was done for each sex. Platelet free plasma (PFP) was collected by a three-step centrifugation process to obtain plasma that is free of platelets. Briefly, blood samples were first centrifuged at 180 g for 11 min at 25 °C to obtain PRP. Then PRP samples were centrifuged at 400 g for 10 min at 25 °C to produce platelet poor plasma (PPP). The PPP samples were transferred into new falcon tubes and underwent a third centrifugation at 400 g for 10 min at 25 °C, for each respective group. The supernatant of the PPP samples was collected and considered as platelet free plasma (PFP). All samples underwent the same centrifugation processes to limit variability. For experiments in resuspended samples, washed platelets were collected from CA exposed mice as described above before they were resuspended with PFP collected from THEC exposed mice (referred to as THEC resuspended) or PFP that is collected from CA exposed mice (referred to as CA resuspended) for control purposes. Subsequentially, all samples were then stimulated with 0.625 µg/mL collagen, and the aggregation and dense granule secretion responses were measured. The % maximal aggregation values were extracted from Aggolink8 software and averaged.

### P-Selectin Expression in Resuspended Platelets

Resuspended samples were prepared as described above. All samples were incubated with 1 mM of calcium chloride, and individually with anti-mouse p-selectin, and incubated for 15 min. Resting platelets were left unstimulated and all other samples were activated with 2.5 µg of the collagen related peptide (CRP), and immediately fixed with 4% paraformaldehyde, and incubated for an additional 15 min. Samples were read using BD Accuri C6 and Cflow plus software (BD Biosciences).

### Cytokine Array

Blood was pooled from 5–8 mice in the CA exposed group or 5–8 mice in the THEC exposed group, from each sex separately, before the PFP samples were prepared as described above, of which 200 µL was used for each array. The Proteome Profiler Mouse XL Cytokine Array was performed as per the manufacturer’s instructions/kit, with a total of 4 arrays; CA males, THEC males, CA females, and THEC females. Each array provided data regarding the abundance of 111 cytokines. Images of the arrays were taken and the results were analyzed using ImageJ software. Each point on the array represents one cytokine, and all points for the 4 arrays were quantified. The data was further analyzed as the ratio of THEC: CA for males and females individually and listed in ascending order for comparison. The data was presented as the top 10% of cytokines that were modulated, i.e., that were either upregulated or downregulated.

### Statistical Analysis

For experiments performed on live mice (i.e., tail bleeding time and thrombosis), and the cotinine assay, at least 5 mice were used per group per sex (CA exposed and THEC exposed) with a single mouse representing each data point. For all other experiments, blood was collected from at least 5 CA exposed and 5 THEC exposed mice of each sex, and pooled prior to experimentation. All experiments were repeated at least 2–3 times (2–3 biological replicates), and each time (for each biological replicate) blood was pooled from at least 5 mice as indicated. Data was analyzed using Graph Pad Prism 7 statistical software (San Diego, CA, USA). Based on the results of a normality test, the data was analyzed as follows; cotinine, tail bleeding time, occlusion time, and blood counts: unpaired t-test; flow cytometry: One-Way ANOVA and Tukey’s multiple comparison; whereas platelet spreading and platelet aggregation and dense granule secretion: Mann–Whitney. Sex analysis for bleeding and occlusion times was analyzed as ΔT and all others were calculated as fold change (ratio of THEC: CA) and analyzed with unpaired t-test. Statistical tests performed were two-tailed, and statistical significance was accepted at p < 0.05, with all data represented as mean ± SEM.

## Results

### Cotinine, the Nicotine Metabolite, is Significantly Elevated in THEC Exposed Mice Regardless of Sex

**Fig. 1:**
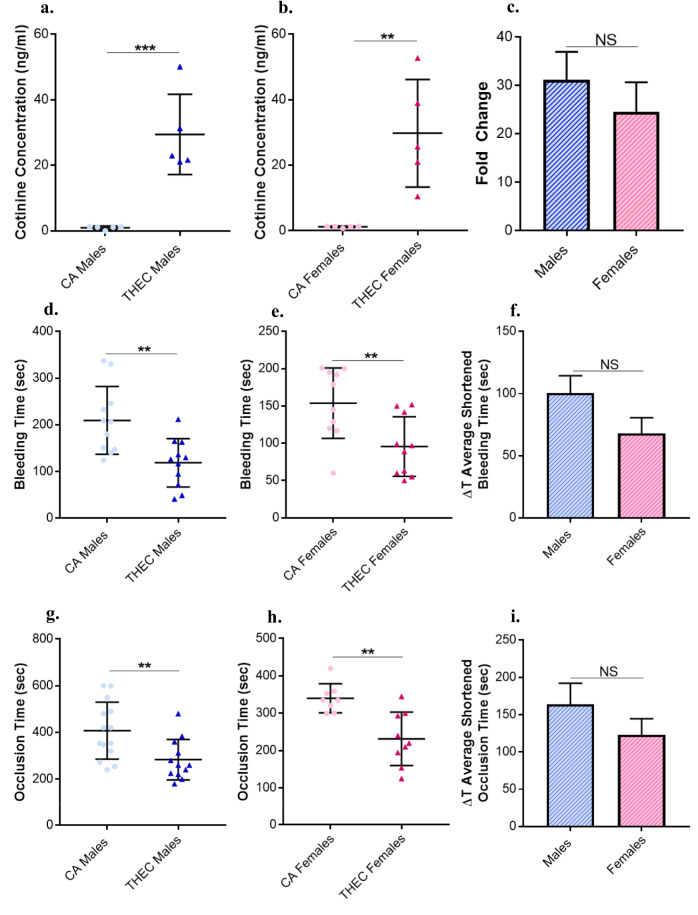
Exposure to THEC increased cotinine concentration and shortened tail bleeding and occlusion times in both male and female mice. **a-b.** Cotinine concentration in the CA exposed and THEC exposed male and female mice was measured post the 4-month exposure period. Each recorded value on the graph illustrates the cotinine concentration of a single mouse (males: CA n = 5, THEC n = 5; females: CA n = 5, THEC n = 5). **c** Fold change of cotinine concentrations in males and females. **d-e.** Tail bleeding times were measured in both males and female mice respectively that were exposed to either CA or THEC. Each recorded value on the graph illustrates the tail bleeding time of one mouse (males: CA n = 11, THEC n = 10; females: CA n = 10, THEC n = 10). **f.** ΔT average shortened bleeding times for males and females as part of the sex dependent analysis. **g-h.** Time to occlusion was recorded for males and females exposed to either CA or THEC, respectively, following carotid artery injury induced by FeCl_3_, and each recorded value is representative of the occlusion time for one mouse (males: CA n = 14, THEC n = 12; females: CA n = 8, THEC n = 9). **i.** ΔT average shortened occlusion timesfor males and females as part of the sex dependent analysis. Data analysis was determined by unpaired t-test (**p < 0.01, ***p < 0.001)

Since cotinine is a major metabolite of nicotine, we measured its concentration to ascertain that THEC toxicants were "delivered" as well as assess the extent of THEC exposure in each of the groups at the conclusion of the 4-month exposure period. As expected, cotinine concentrations were significantly elevated in the THEC exposed mice in both males and females, whereas it was undetectable in the CA exposed mice (Fig. 1a, b). Notably, no sex differences were observed in the cotinine concentration between males and females (Fig. 1c).

### Tail Bleeding and Occlusion Times are Shortened in THEC Exposed Mice Independent of Sex

We first sought to evaluate the role of sex on the hemostatic response of the THEC exposed mice. In order to address this issue, the tail bleeding time assay was performed. As shown in Fig. 1d, a significantly shortened tail bleeding time was observed in the THEC exposed group when compared to the CA exposed group, namely 118.7 ± 15.65 s versus 209.5 ± 21.95 s respectively, in males. Similarly in females, the THEC exposed mice also showed shortened tail bleeding times when compared to the CA exposed, namely, 95.7 ± 12.65 s versus 153.8 ± 14.95 s respectively, as shown in (Fig. 1e). While shortening in bleeding times appears to be more pronounced in the male mice, no statistical significance was observed (Fig. 1f). Next, we conducted the carotid artery FeCl_3_ injury induced thrombosis model to determine the effect of exposure on thrombus formation in both males and females. Similarly, we observed that THEC exposed mice also had significantly shortened occlusion times in comparison to CA exposed, namely, 283.3 ± 25.12 s versus 407.2 ± 32.63 s respectively in males (Fig. 1g) and 231.3 ± 23.78 s versus 340.1 ± 13.8 s respectively in females as shown in Fig. 1h. This suggests increased susceptibility for occlusive CVD in both sexes. To investigate sex-based differences, the ΔT of occlusion times was calculated. However, no differences were observed when comparing males to females (Fig. 1i).

### THEC Exposure Elicits no Significant Changes in Platelet and Peripheral Blood Count in Either Sex

To rule out the influence of blood cell count differences on the THEC prothrombotic phenotype, individual mouse platelet and peripheral blood cells counts were recorded, as displayed in Table 1. Platelet and blood cell counts did not differ significantly between the exposed groups in either sex, thereby eliminating them as a contributing factor to the observed phenotype.

**Table 1:** THEC exposure does not alter platelet or blood cell counts in either males or females

Cell Type	CA Males	THEC Males	*P*-value	CA Females	THEC Females	*P*-Value
Platelets (k/µL)	460 ± 43.84	544.1 ± 80.75	0.3710	481.1 ± 35.87	470.6 ± 18.44	0.8099
MPV (fl)	3.873 ± 0.02371	3.891 ± 0.03149	0.6496	3.973 ± 0.04066	3.9 ± 0.04714	0.2556
RBC (M/µL)	7.298 ± 0.5988	7.765 ± 0.5575	0.5750	8.457 ± 0.2246	7.969 ± 0.4146	0.2906
LY (k/µL)	2.593 ± 0.109	2.885 ± 0.1545	0.1442	2.956 ± 0.166	2.792 ± 0.2724	0.5989
HCT (%)	35.48 ± 3.108	38.21 ± 3.098	0.5413	40.91 ± 1.16	39.24 ± 2.235	0.4944
MO (k/µL)	0.12 ± 0.02255	0.1756 ± 0.01473	0.0557	0.1609 ± 0.01851	0.1356 ± 0.009734	0.2715
WBC (k/µL)	3.252 ± 0.1804	3.734 ± 0.2304	0.1169	3.72 ± 0.1992	3.356 ± 0.3142	0.3233

Platelet and other blood cells counts were recorded from individual mice as described in the methods. *MPV* mean platelet value, *RBC* red blood cells, *LY* lymphocytes, *HCT* hematocrit, *MO* monocytes, *WBC* white blood cells. Unpaired *t*-test was used for statistical analysis between *CA* and THEC, and one-way ANOVA was used for statistical analysis between males and females (data was not found to be significant)

### Platelets from THEC Exposed Mice Exhibit Increased Aggregation and Dense Granule Secretion Independent of Sex

**Fig. 2:**
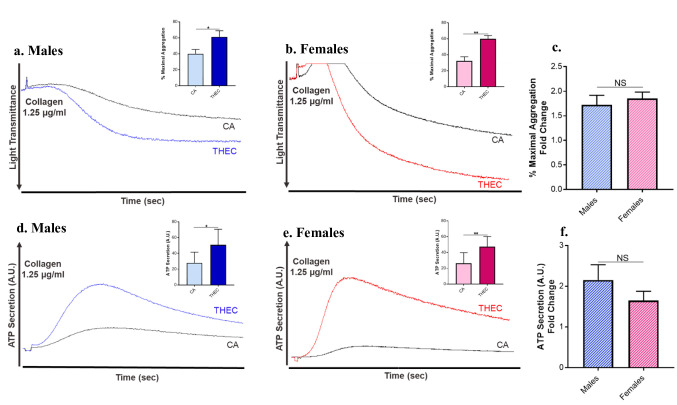
Exposure to THEC enhances platelet function in male and female mice. **a**–**b**. Platelet aggregation was measured and represented by traces along with quantification of % of maximal aggregation (figure inset) in response to 1.25 µg/mL of the collagen agonist in male and female mice, that were exposed to either CA or THEC, respectively. **c**. % maximal aggregation fold change for males and females. **d-e**. Dense granule secretion was measured in platelets from male and female mice, respectively, in response to 1.25 µg/mL of collagen **f**. ATP secretion fold change. Quantification of platelet aggregation and dense granule secretion was analyzed by Mann Whitney (*p < 0.05, **p < 0.01), and fold change by unpaired t-test. Each experiment was repeated 3 times (3 biological replicates), and blood was pooled from at least 5 mice each time/for each of these replicates. **AU:** Arbitrary Units

Since platelets are key players in occlusive CVD, their aggregation response was assessed in CA exposed and THEC exposed mice, following agonist stimulation. It was found that platelets from THEC exposed mice were more hyperactive compared to the CA controls after stimulation with 1.25 µg/mL of collagen in both males and females (Fig. 2a, b). Concerning sex differences, no significant changes in the fold change of maximal aggregation were noticed between male and female exposed mice (Fig. 2c). Similarly, dense granule secretion/ATP secretion was also shown to be enhanced in response to the THEC exposure when platelets were stimulated with 1.25 µg/mL collagen in both sexes (Figs. 2d, e), with no observable differences between males and females (Fig. 2f). These data are consistent with the observed phenotype and suggest that the THEC enhanced risk platelet aggregation/secretion is comparable across the two sexes.

### THEC Promotes Platelet Spreading in a Sex Dependent Manner

**Fig. 3:**
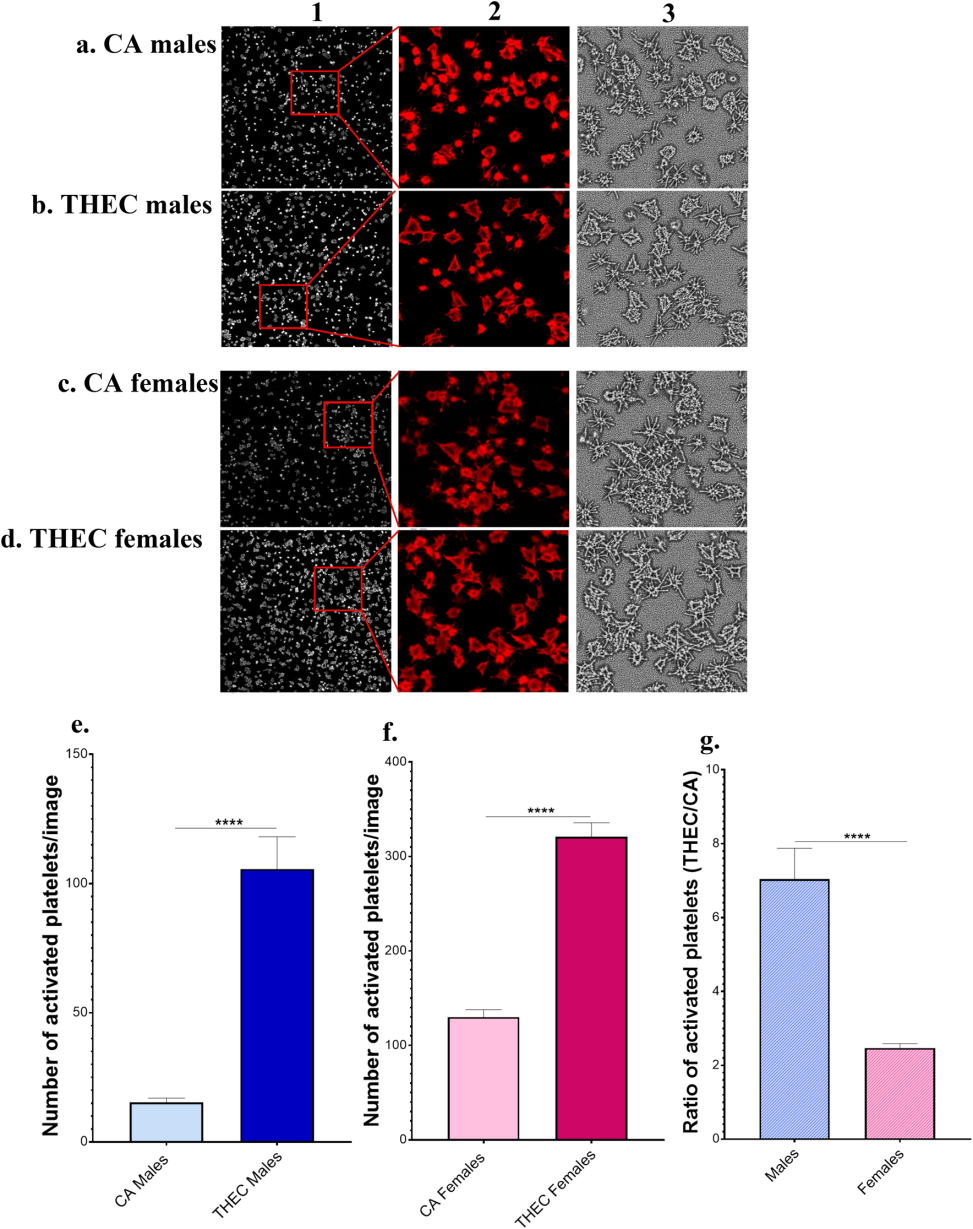
Exposure to THEC enhances platelet spreading in a sex dependent manner. **a–b.** Platelet spreading is assessed in male CA and THEC exposed groups respectively. **c–d.** Platelet spreading is assessed in female CA and THEC exposed groups, respectively. Column 2 represents Zoomed-in sections of the insets highlighted in their respective images. Column 3 represents digitized images generated from column 2 respective images, in ImageJ Software using the Shape Index Map tool. **e–f.** Number of activated platelets per image for CA and THEC males and females respectively,  n = 30 images. **g.** Ratio of THEC: CA activated platelets for males and females. Quantification of activated platelets was assessed by Mann–Whitney and fold change by unpaired t-test (****p < 0.0001). Each experiment was repeated 2 times/2 biological replicates, and blood was pooled from at least 5 mice each time/for each of these replicates. Slides were prepared in triplicates for each group, each time

To further characterize the impact of THEC on platelet activation, we sought to measure the spreading response, in which platelets spread and change their shape through outside-in signaling. Platelet spreading was examined after thrombin stimulation, as depicted in Fig. 3a–d. It was found that there was a significantly higher number of activated platelets in both of the male and female THEC exposed mice, relative to the CA exposed mice when activated with 0.005 U/mL of thrombin (Fig. 3e, f). Interestingly, the ratio of activated platelets between THEC and CA (THEC: CA) was significantly higher in male mice compared to females (Fig. 3g). This finding suggests that some of the THEC effects on platelet function, specifically spreading do manifest in a sex-dependent manner.

### CA Exposed Platelets Resuspended With THEC Platelet Free Plasma Showed Enhanced Aggregation and Dense Granule Secretion

**Fig. 4:**
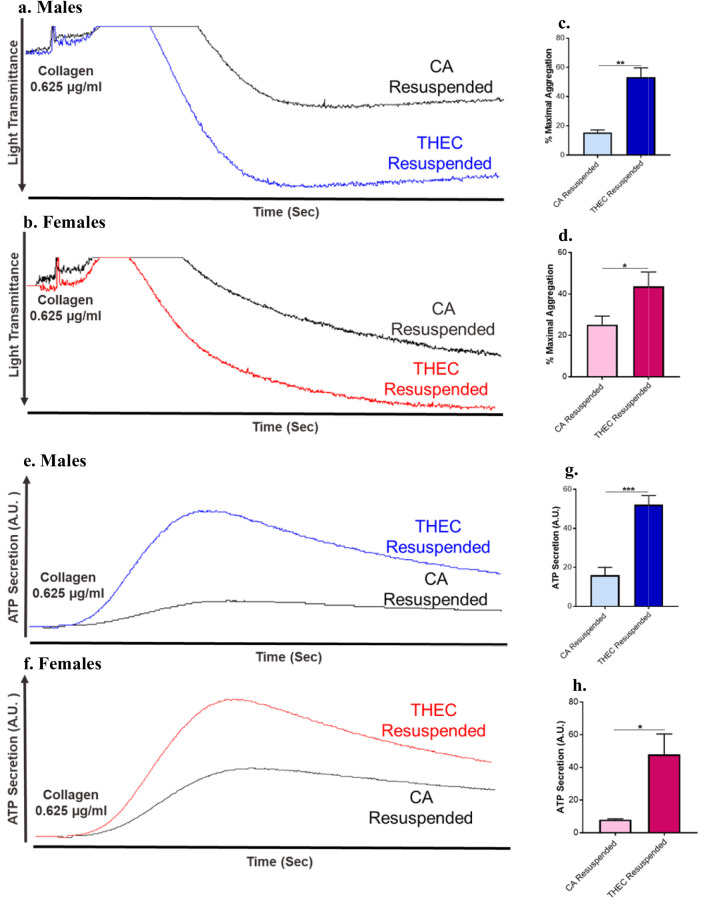
Platelet free plasma from THEC exposed mice enhances aggregation and dense granule secretion in CA platelets. **a–b.** Washed platelets from CA male and female mice were resuspended in platelet free plasma from CA or THEC exposed mice before their aggregation response was measured in response to 0.625 µg/mL of collagen. **c–d.** Quantification of platelet aggregation in male, and female mice, respectively. **e–f.** Washed platelets from CA male and female mice were resuspended in platelet free plasma from CA or THEC exposed mice before their dense granule secretion was measured by determining ATP secretion, in response to 0.625 µg/mL of collagen. **g–h.** Quantification of platelet dense granule secretion in male and female mice, respectively. All statistics for platelet aggregation and dense granule secretion were determined by Mann–Whitney (*p < 0.05, **p < 0.01, ***p < 0.001). Each experiment was repeated 3 times/3 biological replicates, and blood was pooled from at least 5 mice each time/for each of these replicates

To further understand the mechanism by which THEC exposure enhances platelet function, we examined the indirect/systemic effect of THEC on platelets by evaluating the capacity of THEC sera (platelet free sera) to affect the activation of unexposed/CA platelets. Thus, unexposed/CA washed platelets were resuspended/reconstituted in THEC platelet free plasma (referred to as THEC resuspended) or CA platelet free plasma (referred to as CA resuspended) as a control. Notably, when stimulated with collagen, THEC resuspended platelets exhibited significantly enhanced aggregation in both males and females when compared to controls as shown by the representative traces (Fig. 4a, b), with the data quantified in Figs. 4c, d for males and females, respectively. In addition, dense granule secretion was also elevated in the THEC resuspended platelets in both males and females when stimulated with collagen (Fig. 4e–h). These data further suggest the existence of an in direct/systemic effect(s) caused by THEC exposure, thereby impacting platelet function.

### CA Exposed Platelets Resuspended with THEC Platelet Free Plasma Showed Increased p-Selectin Expression

**Fig. 5:**
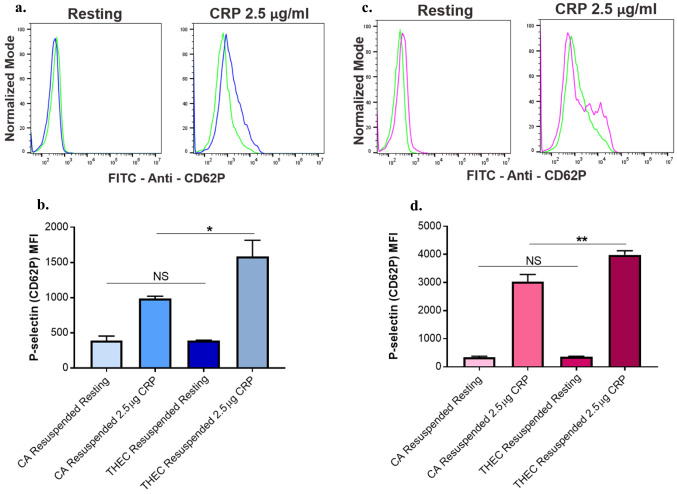
Platelet free plasma from THEC exposed mice enhances p-selectin expression in CA platelets. **a.** Representative histogram of flow cytometric data for washed platelets from CA male mice that were resuspended in platelet free plasma from CA or THEC exposed mice. **b.** Washed platelets from CA male mice were resuspended in platelet free plasma from CA or THEC exposed mice before p-selectin expression was measured after stimulation with 2.5 µg of CRP. **c.** Representative histogram of flow cytometric data for washed platelets from CA female mice that were resuspended in platelet free plasma from CA or THEC exposed mice. **d.** Washed platelets from CA female mice were resuspended in platelet free plasma from CA or THEC exposed mice before P-selectin expression was measured after stimulation with 2.5 µg of CRP. One-Way ANOVA, and Tukey’s multiple comparison post hoc test was conducted for statistical analysis (*p < 0.05, **p < 0.01). Each experiment was repeated 3 times/3 biological replicates, and blood was pooled from at least 5 mice each time for each of these replicates

To further confirm the indirect effects of THEC exposure on platelet function, we examined the surface expression of p-selectin (a measure of platelet alpha granule section) using flow cytometric analysis. When stimulated with a platelet agonist (2.5 µg of CRP), both male and female CA platelets resuspended with THEC showed a significant increase in surface expression of p-selectin, relative to CA resuspension as represented by histograms (Fig. 5a, c) and respective mean fluorescence intensity (Fig. 5b–d), for males and females respectively. Of note, no significant differences in the resting samples were observed for males or females.

### Inflammatory Cytokines are up and Down Regulated in THEC Mice Suggesting a Systemic Effect Caused by Exposure

**Fig. 6:**
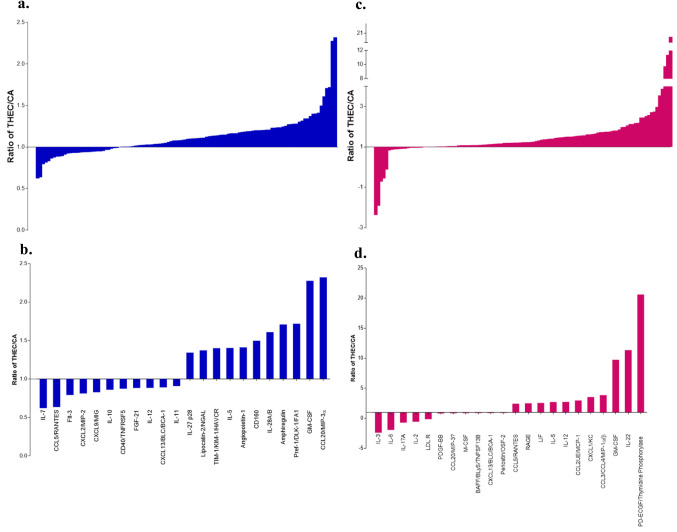
THEC exposure results in up or down regulation of inflammatory cytokines. **a.** Graphical representation of 111 cytokines analyzed in male plasma. **b.** Bar graph representing top 10% upregulated and top 10% downregulated cytokines in male plasma. **c.** Graphical representation of 111 cytokines analyzed in female plasma. **d.** Bar graph representing top 10% upregulated and top 10% downregulated cytokines in female plasma. Cytokines with a ratio > 1 were considered to be upregulated and cytokines with a ratio < 1 were considered to be downregulated. Blood was pooled from at least 5 mice per group, and two technical replicates were repeated for each cytokine

In light of the observed THEC mediated systemic effects on platelets and given that cytokines are also known to modulate platelet function [27], we conducted a high throughput profiling of cytokines in plasma from CA and THEC exposed mice in both males and females. The expression levels of all 111 assessed cytokines are shown in Fig. 6a, c, with the top 10% modulated cytokines (up-, or down-regulated) by exposure displayed in Fig. 6b, d, in males and females, respectively. Interestingly, the THEC exposed females demonstrated a more robust change in the overall expression of cytokines compared to male mice as reflected by THEC/CA ratios. In females, the top 10% of upregulated cytokines included CCL5/RANTES, RAGE, LIF, IL-5, IL-12, CCL2/JE/MCP-1, CXCL1/KC, CCL3/CCL4/MIP-1α/β, GM-CSF, IL-22, and PD-ECGF/Thymidine Phosphorylase; whereas the top 10% downregulated ones included IL-3, IL-6, IL-17A, IL-2, LDL R, PDGF-BB, CCL20/MIP-3, M-CSF, BAFF/Blys/TNFSF13B, CXCL13/BLC/BCA-1, and Periostin/OSF-2 (Fig. 6b). For males, the top 10% upregulated cytokines included IL-27 p28, Lipocalin-2/NGAL, TIM-1/KIM-1/HAVCR, IL-5, Angiopoietin-1, CD160, IL-28A/B, Amphiregulin, Pref-1/DLK-1/FA1, GM-CSF, and CCL20/MIP-3α; whereas the top 10% downregulated ones included IL-7, CCL5/RANTES, Flt-3, CXCL2/MIP-2, CXCL9/MIG, IL-10, CD40/TNFRSF5, FGF-21, IL-12, CXCL13/BCL/BCA-1, IL-11 (Fig. 6d). These data clearly demonstrated that THEC exposure modulates the cytokine profile in plasma of exposed mice in a sex-dependent manner.

## Discussion

Due to the grave consequences of thrombotic CVD and the sex dependent variation in their etiology, identifying unknown risk factors is paramount. This includes assessing the sex-specific effects of the relatively newly recognized/underestimated health threat of THEC exposure. Given the wide variation of e-cig devices and inherent challenges in studying the effects of their thirdhand exposure on human subjects, we sought to utilize a novel rodent exposure protocol/model. This model involved “subjecting” mice to common household materials that are exposed to THEC. The exposure lasted for a four-month period in order to study the sex-dependent effects on platelet function and thrombotic CVDs under chronic exposure conditions. Previous data from our laboratory did establish a prothrombotic phenotype in the THEC exposed mice via direct modulation of platelet function, albeit involved mice of mixed gender [11]. In the current study we determined the role of sex, if any, in THEC-induced thrombosis and platelet hyperactivity. We also examined the systemic impact of THEC exposure on platelet function and inflammatory cytokines, which provides further and mechanistic evidence regarding their deleterious effects in both sexes.

Herein, our novel THEC exposure animal model was again designed to replicate real-life exposure scenarios using a custom e-cig vape chamber to expose standard household materials, which are then used to furnish the animal cages. This ensures that mice do not get directly exposed to e-vape, but rather are subjected to thirdhand exposure. To confirm the validity of our exposures, we quantified cotinine concentrations (the primary metabolite of nicotine; a major component of e-liquid) in the plasma of both exposed and control mice. We observed significant increases of cotinine levels in both male and female mice exposed to THEC at the conclusion of the 4 months exposure window, whereas it was undetectable in the CA mice. Our data did not detect any sex differences in cotinine levels, at least under our exposure conditions- though female mice have been shown to metabolize nicotine at a much faster rate than males [28, 29]; this might be due to the long term nature of our exposure and the cumulative effects over time. These findings not only validate our exposure model but also provide evidence that THEC exposure results in significant systemic amounts of one of the major toxicants emitted by e-vape in both sexes.

We next determined if there are any sex-specific effects in the context of the thrombotic risk, which we have previously shown to manifest in THEC exposed mice [11] to determine if one sex is more susceptible to the detrimental effects than the other by conducting the in vivo tail bleeding and thrombosis model assays. In general, it has been suggested that males are more susceptible to many pathological conditions including CVD [30], and in the case of thromboembolic diseases, males have also shown a higher incidence in humans [30–32]. Furthermore, previous studies have shown that male rats have a higher platelet responsivity, and significantly higher platelet activation in response to stimulation when compared to females [30, 31]. Interestingly, studies have also shown that smoking poses a greater risk of CVD and susceptibility of platelet dysfunction in women compared to men [12, 14]. In terms of the role of sex in e-cig mediated CV harm, it is still poorly studied/understood but remains a critical field of study. In one study that was conducted in mice, it was shown that male mice had higher occurrence of cardiac depression, bradycardia, and bradyarrhythmia that were all induced by e-cig solvents [33]. In contrary, Halstead et al., demonstrated that e-cig use mediated reduction of microvascular endothelial function is more pronounced in females compared to males [16]. Given that nothing is known in the context of THEC, our data serves as the first evidence in this field in the context of the role of sex. Indeed, our data did show that both males and females are prone to THEC enhanced thrombosis, but no sex differences were observed. While no sex differences were observed under our present exposure conditions, it is crucial to highlight the significant increase in thrombosis risk in both sexes, which indicates that THEC seems equally detrimental to both sexes. Interestingly, we have previously shown that females are more sensitive to cigarettes' THS (under one month exposure settings) induced thrombotic events and exhibited more upregulated inflammatory cytokines in comparison to males [26]. While there are some differences in the duration of exposure and reported toxicants profile, our data suggests that THEC may have a distinct sex dependent profile from THS, which will be the subject of future studies. It is important to note that differences in platelet and other blood cell counts were not observed for either sex, which provides conclusive evidence that the observed phenotypes are solely based on the exposure, and not due to any variances of blood cell types between either of the exposure conditions.

Mechanistically, our laboratory has shown that platelets are key players in inducing occlusive arterial thrombus formation in the context of exposure to THS [17, 24, 34] and e-cigs [6, 7], in mice. We have also demonstrated that THEC exposure heightened certain platelet functions, namely aggregation, secretion and integrin activation [11]. However, whether there are sex-dependent THEC related effects remains ill-defined. Although there was no sex difference detected under our current exposure conditions, THEC did significantly enhance platelet aggregation and dense granule secretion in both males and females. This data is consistent with a previous report from one month exposure to THS, in which platelet aggregation and dense granules secretion increased in both males and females to a similar extent [17]. While our data clearly demonstrates that multiple fundamental platelet functional responses (aggregation & secretion) are “disrupted” by THEC, we further explored the mechanism by examining platelet spreading, which is indicative of activation through outside-in signaling of the integrin GPIIb-IIIa. Indeed our data did show a potentiated spreading response, in both males and females. Interestingly, the males exhibited a significantly higher ratio of THEC: CA activated platelets when compared to the females. This data clearly suggests that certain platelet functional responses, like spreading, are more sensitive to THEC exposure in male mice. Conversely, our previous work showed that platelet spreading was more prominent in females compared to males when they were exposed to THS [17]. This finding supports the notion that some modulation of platelet functional responses is not only sex-, but is also tobacco product exposure type-dependent.

While we are the first to establish that “direct” THEC exposure enhances platelet function, we cannot exclude the contribution of a systemic/indirect effect of THEC exposure on platelets. In this connection, it has been well documented that inflammatory cytokines in the plasma are key drivers of platelet activation [35]. It is also evident that direct exposure to tobacco and e-cigs results in proinflammatory cytokines and an oxidative stress environment [5, 36, 37], factors that are known to contribute to the pathophysiology of a host of CVDs [37, 38]. In addition, a recent study has linked thirdhand vaping with pulmonary and systemic inflammatory responses in a murine model [23]. Based on these findings, studying the systemic/indirect effect of THEC exposure on platelet function was imperative, to help gain more insight into the mechanism by which it exerts its occlusive CVD effects. Thus, CA platelets were treated/resuspended with plasma obtained from THEC exposed mice or CA exposed mice as control before measuring platelet aggregation and granule secretion (dense and alpha granules). Interestingly, we observed significant enhancement in platelet aggregation and secretion when the CA platelets were resuspended with THEC plasma from both males and females. This data suggests that plasma exposed to THEC contributes to enhanced platelet activity, thereby supporting the existence of systemic effects. Consequently, we sought to investigate cytokine expression in the plasma of THEC exposed mice, as they were previously shown to contribute to platelets activation [19] and inflammatory mediated thrombosis [39, 40]. It is important to note that even though there are several components in plasma that have the potential to mechanistically contribute to our observed phenotype- which will be the focus of future studies- our primary interest was on inflammatory cytokines. Our cytokine array analysis clearly demonstrated, for the first time, differentially expressed cytokines between the CA exposed and THEC exposed plasma in both male and female mice. Of the multiple cytokines that were found to be upregulated in females, it is worth highlighting thymidine phosphorylase (T.P.), as it was the most upregulated cytokine. It is also known as platelet-derived endothelial cell growth factor and plays an important role in enhancing platelet signaling/function (such as aggregation) and promoting thrombosis given its abundance in platelets [41, 42]. Interestingly, inhibiting this molecule in platelets was shown to produce an antithrombotic effect, indicating that it can also be targeted as a novel antithrombotic therapeutic approach [41]. In males, there were several distinct cytokines that were modulated, with CCL20 being the most highly upregulated one. It is known that CCL20 is produced by epithelial cells and is further expressed under inflammatory conditions [43]. Interestingly and while CCL5/RANTES was upregulated in females, we observed that it was downregulated in males. Though there is not yet a clear explanation for this difference, a study that evaluated RANTES levels in patients with macular degeneration reported similar findings, as they observed lower levels of plasma RANTES in males when compared to females [44]. Despite these differences between males and females, these data provide exciting insight into the systemic proinflammatory effects of THEC exposure and the potential mechanism(s) for indirect effects on platelet function. Beside platelets, these cytokines can also contribute to the observed phenotype through a variety of other mechanisms, including modulation of endothelial cells [45] and other blood cells [46]. Future studies will characterize the detailed mechanism by which THEC mediated systemic inflammatory responses prime platelet reactivity and enhanced thrombosis.

## Conclusion

Taken together, our results collectively support the notion that THEC exposure can result in major negative CV outcomes (i.e., thrombosis and platelet hyperactivity) in both male and female mice, which manifest to a similar extent. We also showed that THEC indirectly exerts sex-dependent effects on platelet function, in part via dysregulated plasma cytokine levels. The similar magnitude of the impact of THEC on both sexes highlights that these devices should not be deemed safe for anyone, including in the context of indirect exposures. We believe our results provide substantial evidence of the negative consequences of e-cigs, especially/including in the form of THEC, which is often underestimated and/or understudied. Thus, limiting the use of e-cig devices in “public areas” is necessary to prevent innocent individuals from being subjected to this ignored health threat.

## Data Availability

Data is provided within the manuscript or supplementary information files.

## References

[1] Goldsborough, E., Osuji, N., & Blaha, M. J. (2022). Assessment of cardiovascular disease risk: A 2022 update. *Endocrinology and Metabolism Clinics of North America,**51*(3), 483–509. 35963625 10.1016/j.ecl.2022.02.005

[2] Willoughby, S., Holmes, A., & Loscalzo, J. (2002). Platelets and cardiovascular disease. *European Journal of Cardiovascular Nursing,**1*(4), 273–288. 14622657 10.1016/s1474-5151(02)00038-5

[3] Martinez Bravo, G., et al. (2024). Platelets in thrombosis and atherosclerosis: A double-edged sword. *The American Journal of Pathology,**194*(9), 1608–1621. 38885926 10.1016/j.ajpath.2024.05.010 PMC11373056

[4] Rose, J. J., et al. (2023). Cardiopulmonary impact of electronic cigarettes and vaping products: A scientific statement from the American Heart Association. *Circulation,**148*(8), 703–728. 37458106 10.1161/CIR.0000000000001160

[5] Alarabi, A. B., et al. (2022). The effect of emerging tobacco related products and their toxic constituents on thrombosis. *Life Sciences,**290*, Article 120255. 34953893 10.1016/j.lfs.2021.120255 PMC9118784

[6] Qasim, H., et al. (2018). Short-Term E-cigarette exposure increases the risk of thrombogenesis and enhances platelet function in mice. *Journal of American Heart Association.,**7*(15), 009264. 10.1161/JAHA.118.009264 PMC6201451 30021806

[7] Ramirez, J. E. M., et al. (2020). The JUUL e-cigarette elevates the risk of thrombosis and potentiates platelet activation. *Journal of Cardiovascular Pharmacology and Therapeutics,**25*(6), 578–586. 32691614 10.1177/1074248420941681 PMC7605053

[8] Goniewicz, M. L., & Lee, L. (2015). Electronic cigarettes are a source of thirdhand exposure to nicotine. *Nicotine & Tobacco Research,**17*(2), 256–258. 25173774 10.1093/ntr/ntu152 PMC4837997

[9] Sakamaki-Ching, S., et al. (2022). Dermal thirdhand smoke exposure induces oxidative damage, initiates skin inflammatory markers, and adversely alters the human plasma proteome. *eBioMedicine,**84*, Article 104256. 36137411 10.1016/j.ebiom.2022.104256 PMC9494172

[10] Yeh, K., et al. (2022). Thirdhand smoke from tobacco, e-cigarettes, cannabis, methamphetamine and cocaine: Partitioning, reactive fate, and human exposure in indoor environments. *Environment International,**160*, Article 107063. 34954646 10.1016/j.envint.2021.107063

[11] Umphres, S. S., et al. (2024). Investigation of the impact of thirdhand e-cigarette exposure on platelet function: A pre-clinical study. *Tobacco Induced Diseases,**22*, 10–18332. 10.18332/tid/185286 PMC10980912 38560550

[12] Gaalema, D. E., et al. (2024). Differential effects of cigarette smoking on cardiovascular disease in females: A narrative review and call to action. *Preventive Medicine,**188*, Article 108013. 38815766 10.1016/j.ypmed.2024.108013

[13] Alam, F., & Silveyra, P. (2023). Sex differences in E-cigarette use and related health effects. *International Journal of Environmental Research and Public Health*. 10.3390/ijerph20227079 37998310 10.3390/ijerph20227079 PMC10671806

[14] Butkiewicz, A. M., et al. (2006). Does smoking affect thrombocytopoiesis and platelet activation in women and men? *Advances in Medical Sciences,**51*, 123–126. 17357291

[15] Schirone, L., et al. (2022). Sex-related differences in oxidative, platelet, and vascular function in chronic users of heat-not-burn vs. traditional combustion cigarettes. *Antioxidants (Basel)*. 10.3390/antiox11071237 35883727 10.3390/antiox11071237 PMC9311916

[16] Halstead, K. M., et al. (2023). Sex differences in oxidative stress-mediated reductions in microvascular endothelial function in young adult e-cigarette users. *Hypertension,**80*(12), 2641–2649. 37800370 10.1161/HYPERTENSIONAHA.123.21684 PMC10848654

[17] Qadri, S., et al. (2024). Sex dependent occlusive cardiovascular disease effects of short-term thirdhand smoke exposure. *Nicotine & Tobacco Research*. 10.1093/ntr/ntae061 10.1093/ntr/ntae061 PMC11339167 38520288

[18] Ali, H. E. A., et al. (2022). In utero thirdhand smoke exposure modulates platelet function in a sex-dependent manner. *Haematologica,**107*(1), 312–315. 34525795 10.3324/haematol.2021.279388 PMC8719073

[19] Khodadi, E. (2020). Platelet function in cardiovascular disease: Activation of molecules and activation by molecules. *Cardiovascular Toxicology,**20*(1), 1–10. 31784932 10.1007/s12012-019-09555-4

[20] Makena, P., et al. (2023). Biomarkers of exposure and potential harm in two weeks of smoking abstinence: Changes in biomarkers of platelet function, oxidative stress, and inflammation. *International Journal of Molecular Sciences*. 10.3390/ijms24076286 37047257 10.3390/ijms24076286 PMC10093936

[21] Hahad, O., et al. (2023). Tobacco smoking and vascular biology and function: Evidence from human studies. *Pflugers Archiv. European Journal of Physiology,**475*(7), 797–805. 36961561 10.1007/s00424-023-02805-z PMC10264470

[22] Elisia, I., et al. (2020). The effect of smoking on chronic inflammation, immune function and blood cell composition. *Scientific Reports,**10*(1), Article 19480. 33173057 10.1038/s41598-020-76556-7 PMC7655856

[23] Commodore, S., et al. (2023). Thirdhand vaping exposures are associated with pulmonary and systemic inflammation in a mouse model. *Journal of Environmental Exposure Assessment.,**2*(4), 22. 38741701 10.20517/jeea.2023.27 PMC11090496

[24] Villalobos-García, D., et al. (2022). Exposure of mice to thirdhand smoke modulates in vitro and in vivo platelet responses. *International Journal of Molecular Sciences*. 10.3390/ijms23105595 35628405 10.3390/ijms23105595 PMC9144272

[25] Alarabi, A. B., et al. (2020). The G-protein βγ subunits regulate platelet function. *Life Sciences,**262*, Article 118481. 32971104 10.1016/j.lfs.2020.118481

[26] Qadri, S., et al. (2024). Sex-dependent occlusive cardiovascular disease effects of short-term thirdhand smoke exposure. *Nicotine & Tobacco Research,**26*(9), 1225–1233. 38520288 10.1093/ntr/ntae061 PMC11339167

[27] Chen, Y., et al. (2020). Role of platelet biomarkers in inflammatory response. *Biomarker Research,**8*(1), 28. 32774856 10.1186/s40364-020-00207-2 PMC7397646

[28] Rubinstein, M. L., et al. (2013). Race, gender, and nicotine metabolism in adolescent smokers. *Nicotine & Tobacco Research,**15*(7), 1311–1315. 23239845 10.1093/ntr/nts272 PMC3682846

[29] Nguyen, K., et al. (2020). The impact of sex on changes in plasma corticosterone and cotinine levels induced by nicotine in C57BL/6J mice. *Brain Sciences*. 10.3390/brainsci10100705 33023022 10.3390/brainsci10100705 PMC7601418

[30] Johnson, M., Ramey, E., & Ramwell, P. W. (1975). Sex and age differences in human platelet aggregation. *Nature,**253*(5490), 355–357. 1110780 10.1038/253355a0

[31] Johnson, M., & Ramwell, P. (1974). Androgen mediated sex differences in platelet aggregation. *The Physiologist,**17*(256), 355–357.

[32] Acheson, J., Danta, G., & Hutchinson, E. C. (1972). Platelet adhesiveness in patients with cerebral vascular disease. *Atherosclerosis,**15*(1), 123–127. 5013275 10.1016/0021-9150(72)90045-7

[33] Carll, A. P., et al. (2022). E-cigarettes and their lone constituents induce cardiac arrhythmia and conduction defects in mice. *Nature Communications,**13*(1), 6088. 36284091 10.1038/s41467-022-33203-1 PMC9596490

[34] Karim, Z. A., et al. (2015). Third-hand smoke: Impact on hemostasis and thrombogenesis. *Journal of Cardiovascular Pharmacology*. 10.1097/FJC.0000000000000260 25853992 10.1097/FJC.0000000000000260

[35] Theofilis, P., et al. (2021). Inflammatory mediators of platelet activation: Focus on atherosclerosis and COVID-19. *International Journal of Molecular Sciences*. 10.3390/ijms222011170 34681830 10.3390/ijms222011170 PMC8539848

[36] Prasad, K. N., & Bondy, S. C. (2022). Electronic cigarette aerosol increases the risk of organ dysfunction by enhancing oxidative stress and inflammation. *Drug and Chemical Toxicology,**45*(6), 2561–2567. 34474637 10.1080/01480545.2021.1972680

[37] Emma, R., et al. (2022). The impact of tobacco cigarettes, vaping products and tobacco heating products on oxidative stress. *Antioxidants*. 10.3390/antiox11091829 36139904 10.3390/antiox11091829 PMC9495690

[38] Niemann, B., et al. (2017). Oxidative stress and cardiovascular risk: obesity, diabetes, smoking, and pollution: Part 3 of a 3-part series. *Journal of the American College of Cardiology,**70*(2), 230–251. 28683970 10.1016/j.jacc.2017.05.043 PMC5568826

[39] Snijders, A. M., et al. (2021). In utero and early-life exposure to thirdhand smoke causes profound changes to the immune system. *Clinical Science (London, England),**135*(8), 1053–1063. 10.1042/CS20201498 PMC8086195 33851706

[40] Walsh, T. G., Harper, M. T., & Poole, A. W. (2015). SDF-1α is a novel autocrine activator of platelets operating through its receptor CXCR4. *Cellular Signalling,**27*(1), 37–46. 25283599 10.1016/j.cellsig.2014.09.021 PMC4265729

[41] Li, W., et al. (2014). Thymidine phosphorylase participates in platelet signaling and promotes thrombosis. *Circulation Research,**115*(12), 997–1006. 25287063 10.1161/CIRCRESAHA.115.304591 PMC4258140

[42] Li, W., & Yue, H. (2018). Thymidine phosphorylase: A potential new target for treating cardiovascular disease. *Trends in Cardiovascular Medicine,**28*(3), 157–171. 29108898 10.1016/j.tcm.2017.10.003 PMC5856583

[43] Hamed, A. M., et al. (2023). Serum CCL20: A novel potential marker of cardiovascular risk in alopecia areata patients. *Journal of the Egyptian Women’s Dermatologic Society*. 10.4103/jewd.jewd_27_23

[44] Fonteh, C. N., et al. (2022). Sex differences in RANTES (CCL5) in patients with intermediate age-related macular degeneration (AMD) and controls with no AMD. *Translation Vision Science Technology,**11*(2), 12. 10.1167/tvst.11.2.12 PMC8842629 35133404

[45] Groten, S. A., et al. (2023). Multi-omics delineation of cytokine-induced endothelial inflammatory states. *Communications Biology,**6*(1), 525. 37188730 10.1038/s42003-023-04897-w PMC10184633

[46] Karsten, E., Breen, E., & Herbert, B. R. (2018). Red blood cells are dynamic reservoirs of cytokines. *Scientific Reports,**8*(1), 3101. 29449599 10.1038/s41598-018-21387-w PMC5814557

